# Therapeutics effects of bovine colostrum applications on gastrointestinal diseases: a systematic review

**DOI:** 10.1186/s13643-024-02489-1

**Published:** 2024-02-26

**Authors:** Parisa Hajihashemi, Fahimeh Haghighatdoost, Nazila Kassaian, Marzieh Rahim Khorasani, Laleh Hoveida, Hassan Nili, Babak Tamizifar, Peyman Adibi

**Affiliations:** 1 Isfahan Gastroenterology and Hepatology Research Center, Isfahan University of Medical Sciences, Isfahan, Iran; 2 Isfahan Cardiovascular Research Center, Cardiovascular Research Institute, Isfahan University of Medical Sciences, Isfahan, Iran; 3 Nosocomial Infection Research Center, Isfahan University of Medical Sciences, Isfahan, Iran; 4 Infectious Diseases and Tropical Medicine Research Center, Isfahan University of Medical Sciences, Isfahan, 8174673461 Iran; 5 Department of Microbiology, Falavarjan Branch, Islamic Azad University, Isfahan, Iran; 6 Zeitoon Isfahan Vaccine Innovators Company, Isfahan Scince and Technology Town, Isfahan, Iran

**Keywords:** Bovine colostrum, Gastrointestinal diseases, Diarrhea, Abdominal pain

## Abstract

**Background:**

Evidence on the effects of bovine colostrum (BC) supplementation on gastrointestinal (GI) diseases is conflicting.

**Objectives:**

This systematic review summarized the findings of clinical trials (CTs) on the effects of BC supplementation on GI diseases.

**Methods:**

A systematic search was conducted in online databases, including PubMed, ISI Web of Science, and Scopus, until March 2021 and updated until December 2023. CTs investigated BC’s effect on any measurable symptomatic change in terms of GI health as the primary outcome variable or as one of the outcomes in any population eligible for this systematic review.

**Results:**

Out of 6881 records, 22 CTs (uncontrolled = 4, cross-over = 1, and parallel = 17) with 1427 patients were enrolled in the systematic review. Diarrhea, the most frequently evaluated symptom (20 interventional arms), was decreased in frequency with BC supplementation in 15 of these arms. However, most studies reported no change in its duration. BC supplementation consistently reduced stool frequency across all seven studies. Abdominal pain relief was noted in four interventional arms but showed no improvement in five others. Assessment of other GI symptoms was limited, yielding inconclusive results.

**Conclusions:**

There is limited evidence on the effects of BC on GI diseases, with mixed findings. More well-designed controlled clinical trials are required to explore its effects.

**Supplementary Information:**

The online version contains supplementary material available at 10.1186/s13643-024-02489-1.

## Introduction

Gastrointestinal (GI) diseases, which significantly impact global health, affect the GI tract from the mouth to the anus [1]. GI diseases can be categorized as either functional, which are not accompanied by visible structural changes, or structural, such as inflammatory bowel disease (IBD), where both function and appearance of the GI tract are affected [2]. On the other hand, functional GI diseases are characterized by symptoms (including pain, constipation, nausea, bloating, and diarrhea) without any apparent structural changes to the GI tract [3]. GI diseases are among the most common reasons people seek medical care [4], and are typically caused by infections, unhealthy diet, stress, and medications’ side effects [2]. Given the varied causes of GI diseases, there is a growing interest in diverse treatment approaches, including dietary modifications [5, 6]. Dietary approaches are recognized as a novel alternative treatment option for managing of GI diseases [5, 6].

Recently, the therapeutic potential of colostrum in promoting gut health has garnered significant attention [7, 8]. Colostrum is the primary milk secretion of the mammary gland produced by mammals after parturition [9]. Its composition differs from the milk that is subsequently produced. Colostrum contains a higher concentration of fat, protein, peptides, immunoglobulins, vitamins, minerals, hormones, antimicrobial peptides (e.g., lactoferrin, lactoperoxidase), and growth factors, and lower concentration of lactose compared to mature milk [10]. Therefore, the additional benefit of colostrum in the prevention and treatment of GI diseases may be attributed to its higher concentration of immunoglobulins and antimicrobial factors than mature milk [8]. Similarly, bovine colostrum (BC) is a rich source of nutrients and immunological agents [9]. While BC is essential for the nutrition, growth, and development of newborn calves’ GI tract and immune system, its potential therapeutic applications in humans are also being explored [11, 12]. To date, BC has been investigated for several GI diseases [8, 13]. Preventing the effects of BC on intestinal permeability in healthy individuals and patients has been indicated in a recent systematic review [9]. Moreover, a recent meta-analysis demonstrated the effectiveness of BC in reducing the frequency and alleviating symptoms of childhood infectious diarrhea [14]. Despite multiple clinical trials (CTs) evaluating BC’s impact on GI diseases [15–17], there still needs to be a consensus on its efficacy [16, 18], while others reported no significant benefits [19, 20]. Recognizing this gap in the literature, our study aims to systematically review the current evidence on BC’s effects on GI diseases.

## Methods

This systematic review was written with the preferred reporting items for systematic reviews and meta-analyses (PRISMA) guidelines [21].

### Search strategy

The online databases including PubMed, ISI Web of Science and Scopus were searched systematically up to March 2021 to find relevant publications. We used combinations of the following search terms: (colostrum[all] OR colostrums[all] OR bovine[all] OR cow[all] OR cows[a ll] OR cattle[all]) AND ((Disease[all] AND Gastrointestinal[all]) OR (Diseases[all] AND Gastrointestinal[all]) OR “Gastrointestinal Disease*”[all] OR “Gastrointestinal Disorders”[all] OR “Gastrointestinal Disorder”[all] OR “Functional Gastrointestinal Disorders”[all] OR “Functional Gastrointestinal Disorder”[all] OR (“Gastrointestinal Disorder”[all] AND Functional[all]) OR (“Gastrointestinal Disorders”[all] AND Functional[all])). The complete search strategy is shown in Additional file 1. The reference lists of the retrieved articles were also hand-searched for additional relevant studies. No time or language limitation was applied, and the search was updated until December 2023 using PubMed’s e-mail alert service.

### Study selection

Relevant studies were identified based on our PICOS criteria (patients, intervention, comparator, outcome, and study design). Studies with the following criteria were selected: (1) randomized controlled trials (RCTs) or other CTs; (2) being conducted on any population (infants, pediatrics, adults aged, sick and healthy subjects); (3) considering BC or hyperimmune bovine colostrum (HBC) or mixed BC product as intervention; (4) considering no control group or any intervention as a control group; (5) measuring any type of symptomatic change in gastrointestinal health as the primary outcome variable or as one of the outcomes. Studies were excluded if they were non-original (commentaries, editorials, or reviews), animal or in vitro experimental studies, or used non-bovine colostrum (e.g., human colostrum). Unpublished studies or gray literature were also excluded from the current review. Moreover, when a study was performed on separate groups of participants, data of each group compared to the control group were considered an independent study. Two independent authors (PH, LH) screened the retrieved articles using our search strategy to identify potentially eligible studies. Titles and abstracts of articles were reviewed to decide which articles were relevant. Then, full texts of identified articles were reviewed to assess their eligibility based on predefined inclusion and exclusion criteria. Any disagreements were resolved in consultation with the principal investigator (PA).

### Data extraction

Two reviewers (PH, LH) independently extracted the following information from each study: first author’s last name; publication date; trial design (single arm/parallel/cross-over); country of origin; mean age or age range of participants; sex of participants; sample size, number of individuals in intervention and control groups, duration of intervention, intervention and control diet, side effects, and outcomes assessed.

### Quality assessment

At least two independent members (PH, FH) critically appraised each study. They assessed the risk of bias methodology using Cochrane Collaboration Risk of Bias Tool (ROB) [22] based on the following domains: random sequence generation (selection bias), allocation concealment (selection bias), blinding of participants, personnel (performance bias), and outcome assessors (detection bias), incomplete outcome data (attrition bias), selective outcome reporting (reporting bias), and other sources of bias. According to the Cochrane Handbook, the judgment of each domain was done using the terms “Low,” “High,” or “Unclear” risk of bias. Low risk of bias interpreted as plausible bias unlikely to seriously alter the results. Unclear risk of bias interpreted as plausible bias that raises some doubt about the results. High risk of bias interpreted as plausible bias that seriously weakens confidence in the results. Any discrepancies were resolved by discussion.

## Results

### Search results and study selection

**Fig. 1:**
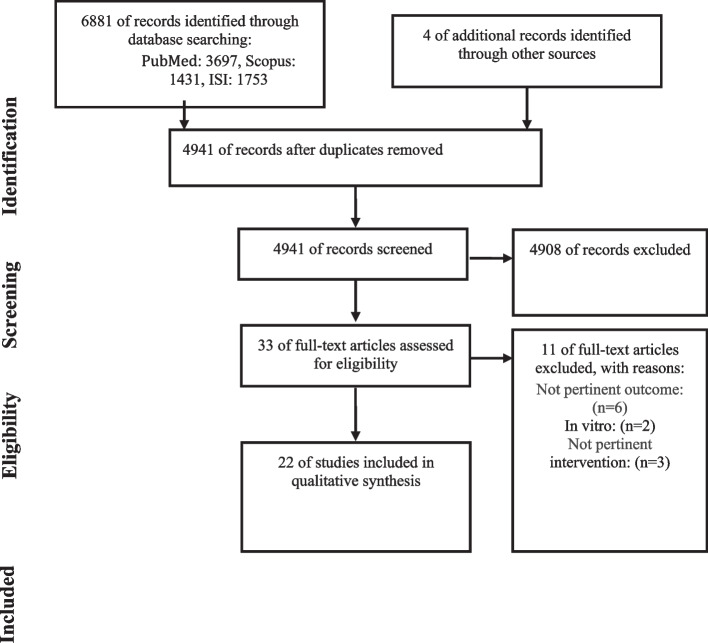
Flow chart of studies reviewed

A total of 6881 records were identified through the initial search. After removing duplicates (*n* = 1940) and screening titles and abstracts, 33 articles remained for further evaluation. The full texts of these articles were read by two independent reviewers (PH and MR) to assess their eligibility, leading to the exclusion of 11 articles. These exclusions were due to the following reasons: non-relevant outcome (*n* = 6), in vitro studies (*n* = 2), and non-relevant intervention (*n* = 3) [23–33]. In order to avoid missing any relevant articles, the reference lists of identified eligible studies were assessed. Hand-searching these articles resulted in identifying *n* = 4 additional studies. Among these articles, 22 met our inclusion criteria and were enrolled [15–20, 24–39] (Fig. 1).

### Study characteristics

Table 1 shows the characteristics of eligible studies for inclusion in the present systematic review. Two studies [29, 36] prescribed BC in intervention and control arms. In the study by Sanctuar et al. [36], participants in the intervention group consumed BC besides probiotics, while those in the control group consumed BC alone. In the other one, the effect of HBC with non-immunized BC was compared [34]. Of the 22 relevant articles (with a total of 31 effect sizes), four were uncontrolled [18, 25, 31, 32], one was cross-over [35], and the remaining 17 studies were parallel RCTs [15–17, 19, 20, 24, 26–30, 33–35, 37–39]. Seven studies (with ten effect sizes) were conducted in Asia [17, 18, 20, 26, 28, 29, 35], six studies (with seven effect sizes) in Europe [16, 27, 31, 32, 34], two studies (with five effect sizes) in Australia [15, 33], three studies in the US [30, 36, 39], and four studies in Africa [24, 25, 37, 38]. Two studies comprised only men [28, 31] and others, both men and women [15–20, 24–27, 29, 30, 32–39]. Of these 22 articles, 14 studies were conducted among infants and children [18–20, 26–29, 33–39], six studies among adults [15, 17, 24, 25, 30, 31], and two studies among both children and adults [16, 32]. Except for four studies on healthy subjects [15, 26, 30, 37], others were conducted on patients with diarrhea, AIDS, *H. pylori*, colitis, or hospitalized in intensive care unit (ICU) or deficient birth weight infants. The intervention duration ranged from 3 days [39, 40] to 3 months [41]. Patients in the intervention arm received either BC or HBC with a range of less than 1 [15, 36] to 100 g/d [42]. In all trials, oral BC was prescribed apart from one that used an enema supplement [16]. BC was prescribed solely in most of the studies, but in some studies, it was along with other interventions [24, 36–39]. In the control group, participants received conventional treatments or market milk or bovine serum albumin. The GI symptoms evaluated in these studies included diarrhea and bowel movement, the frequency and presence of bacterial pathogens in stool, *H. pylori* infection, abdominal pain, rectal bleeding, nausea, abdominal tenderness, abdominal distension, vomiting, pre-feed significant gastric residuals, constipation, gas frequency, and gut microbiota.

**Table 1:** Characteristics of studies included in the current systematic review

**Authors with year of publication**	**Study design/ study location**	**Participant (treatment/control) and sex**	**Age range/mean age (y)**	**Subjects**	**Diet type**		**Duration (month/wk/day)**	**Outcomes**	**Side effects**	**Conclusion**
					**Intervention**	**Control**				
**Ebina et al. ** [39]. **1985**	Parallel RCT Location: Japan	44 N (18 T/26 C) (24 M/20 F)	3 months–6 years	Infants with acute diarrhea and rotavirus infection	HBC (20–50 ml/d)	Market milk	3 days	Diarrhea, bowel movements, and virus shedding in stool	None	HBC significantly prevented the incidence of diarrhea caused by rotavirus (17% vs.86%; *p* < 0.05) but had no effect on duration of diarrhea, bowel movements, or virus shedding in stool
**Zadnikova et al. ** [28] **1987**	Parallel RCT Location: Prague	46 N (32 T/14 C)	n.d	Premature infants with diarrhea	BC antibodies (6 times daily)	Conventional treatment (i.e., diet, rehydratation, and antibiotics)	5 days	Number and quality of stool and presence of bacterial pathogens in stool	None	BC antibodies group had a significantly lower frequency of bacterial pathogens in stools (28% vs. 80%; *p* < 0.05), but BC antibodies had no effect on number and quality of stool
**Zadnikova et al. ** [28] **1987**	Parallel RCT Location: Prague	39 N (24 T/15 C)	n.d	Full-term infants with diarrhea	BC antibodies (6 times daily)	Conventional treatment (i.e., diet, rehydratation, and antibiotics)	5 days	Number and quality of stool and presence of bacterial pathogens in stool	None	BC antibodies group had a significantly lower frequency of bacterial pathogens in stools (30% vs. 80%; *p* < 0.05), but BC antibodies had no effect on number and quality of stool
**Davidson et al. ** [43], **1989**	Parallel RCT Location: Australia	120 N (55 T/65 C)	3–15 months	Infants admitted to the hospital	HBC (50 ml/d)	Infant formula	10 days	Rotavirus diarrhea and length of hospital stay	None	HBC protected susceptible children against rotavirus infection (100% vs. 86%; *p* < 0.01), but the mean length of hospital stay was similar in both groups (HBC 4.4 days, control 3.4 days)
**Rump et al. ** [36], **1992**	Single-arm pilot CT Location: Germany	37 N (31 M/6 F)	1–54	Patients with AIDS-associated chronic diarrhea	BCI (10 g/day)	-	10 days	Frequency and duration of diarrhea and stool pathogens	Nausea and flatulence	BCI decreased frequency and quantity of diarrhea in 76% of patients, stool pathogens disappeared following BCI therapy, and diarrhea recurred in 32.4% of patients in the first 10 days after the end of therapy
**Plettenberg et al. ** [37], **1993**	Single-arm CT Location: Germany	18 N (18 M)	26–58	HIV-positive patients with chronic diarrhea	BCI (10 g/d)	-	10 days	Stool frequency	None	BCI led to complete (40%) or partial remission (reduction in the frequency of diarrhea ≤ 50%) (24%) of diarrhea
**Okhuysen et al. ** [44], **1998**	Parallel RCT Location: US	16 N (5 T/6 C)	18–45	Healthy subjects challenged with *Cryptosporidium parvum*	BACI (10 g three times a day)	Nonfat milk	5 days	Diarrhea	None	HBC was associated with a trend toward less diarrhea in comparison with placebo group (− 36% vs. 11%; *p* = 0.08)
**Okhuysen et al. ** [44], **1998**	Parallel RCT Location: US	16 N (5 T/6 C)	18–45	Healthy subjects challenged with *Cryptosporidium parvum*	Reinforced BACI (20 g three times a day)	Nonfat milk	5 days	Diarrhea	None	Reinforced BACI had no significant effect on diarrhea
**Casswall et al. ** [34], **1998**	Parallel RCT Location: Bangladesh	24 N (12 T/12 C)	4–29 month	*H. pylori*-positive infants	HBCI (1 g/day)	Non-immunized BC (1 g/day)	1 month	*H. pylori* infection	n.d	HBCI did not eradicate *H. pylori* infection in infants
**Sarker et al. ** [45], **1998**	Parallel RCT Location: Bangladesh	80 N (40 T/40 C) (80 M)	4–24 month	Children with rotavirus diarrhea	HBCI (10 g/d)	Milk powder	4 days	Stool output, stool frequency, duration of diarrhea, and presence of rotavirus in stool	None	HBCI significantly reduced stool output, stool frequency, and total duration of diarrhea (*p* < 0.05) and resulted in greater recovery (number, 33 vs. 21; *p* = 0.001) and earlier clearance of rotavirus from stool (mean day, 1.5 vs. 2.9; *p* < 0.001)
**Huppertz et al. ** [46], **1999**	Parallel RCT Location: Germany	27 N (13 T/14 C) (13 M/14 F)	1 month–18 years	Children with diarrhea caused by *E. coli*	BC (7 g three times a day)	Gelatin	14 days	Stool frequency	None	BC significantly reduced stool frequencies (mean reduction, 2 ± 2 vs. 1 ± 3; *p* = 0.027)
**Khan et al. ** [16], **2002**	Parallel RCT Location: UK	14 N (8 T/6 C) (8 F/6 M)	16–75	Patients with mild to moderately severe distal colitis	BC enema (100 ml twice daily) + mesalazine (1.6 g ⁄ day)	Bovine serum albumin + mesalazine (1.6 g ⁄ day)	4 wks	Bowel symptoms: patient well-being, abdominal pain, rectal bleeding, anorexia ⁄ nausea, bowel frequency, stool consistency, and abdominal tenderness	None	BC enema significantly improved bowel symptoms score (mean change, − 2.9 (95% CI; − 0.3, 5.4) vs. 0.5 (95% CI; 2.4, + 3.4)) and inflammation
**Tawfeek et al. ** [47], **2003**	Parallel RCT Location: Iraq	59 N (30 T/29 C) (30 M/29 F)	n.d	Healthy infants	Standard formula plus HBCI (polyvalent) (0.5 g/kg/d)	Milk formula without immunoglobulin	7 days	Diarrheal morbidity and isolation of *E.coli* in stool	None	HBCI supplementation was associated with reduction in diarrheal morbidity (a lower incidence of diarrhea (1.9 ± 1.1 vs. 3.5 ± 2.6; *p* < 0.01), lower number of stools per day (3.3 ± 1.3 vs. 6.6 ± 1.4; *p* < 0.01), and shorter duration of diarrhea (4.5 ± 3.6 vs. 6.5 ± 4.3; *p* < 0.01)) The isolation of *E. coli* was positive in 14% of stool cultures in HBCI and 50% in control group
**Tawfeek et al. ** [47],** 2003**	Parallel RCT Location: Iraq	54 N (25 T/29 C) (28 M/26 F)	n.d	Healthy infants	Standard formula plus HBCI (monovalent) (0.5 g/kg/d)	Milk formula without immunoglobulin	7 days	Diarrheal morbidity and isolation of *E.coli* in stool	None	HBCI supplementation had no significant effect on incidence of diarrhea and duration of diarrhea The isolation of *E. coli* was positive in 40% of stool cultures in HBCI and 50% in control group
**Tawfeek et al. ** [47],** 2003**	Parallel RCT Location: Iraq	52 N (23 T/29 C) (25 M/27 F)	n.d	Healthy infants	Standard formula plus BCI (0.5 g/kg/d)	Milk formula without immunoglobulin concentrate supplementation	7 days	Diarrheal morbidity and isolation of *E.coli* in stool	None	BCI supplementation had no significant effect on incidence and duration of diarrhea The isolation of *E. coli* was positive in 46% of stool cultures in HBCI and 50% in control group
**Florén et al. ** [38],** 2006**	Single-arm CT Location: Nigeria	30 N (15 M/15 F)	20–56	Patients with HIV-associated diarrhea	BCP (50 g two times a day)	-	4 wks	Stool evacuations	None	BC decreased stool evacuations per day (7.09 ± 2.7 to 1.39 ± 0.5; *p* < 0.01)
**Kaducu et al. ** [42],** 2011**	Parallel RCT Location: Northern Uganda	87 N (45 T/42 C) (60 F/27 M)	≥ 18	Patients with HIV-associated diarrhea	BC (50 g twice a day) + standard anti-diarrhea treatment	Standard anti-diarrhea treatment	4 wks	Daily stool frequency	n.d	BC significantly decreased daily stool frequency (79% vs. 58%; *p* < 0.001)
**Otto et al. ** [15], **2011**	Parallel RCT Location: Australia	30 N (15 T/15 C)	18–40	Healthy adults	HBC with sodium bicarbonate (400 mg three times a day)	Lactose	7 days	Diarrhea, abdominal pain, and isolation of *E. coli* in stool	None	HBC was significantly effective in protecting against the development of diarrhea caused by ETEC (volunteers with diarrhea, 7% vs.73%; *p* = 0.0005) and lowering abdominal pain (0% vs. 33%;*p* = 0.04), but HBC had no significant effect on the number of diarrheal stools and the viability of *E.coli*
**Otto et al. ** [15],** 2011**	Parallel RCT Location: Australia	29 N (14 T/15 C)	18–40	Healthy adults	HBC without sodium bicarbonate (200 mg three times a day)	Lactose	7 days	Diarrhea, abdominal pain, and isolation of *E.coli* in stool	None	HBC was significantly effective in protecting against the development of diarrhea caused by ETEC (volunteers with diarrhea, 36% vs. 86%; *p* = 0.02) and lowering abdominal pain (14% vs. 36%;*p* = 0.04), but HBC had no significant effect on the number of diarrheal stools and the viability of *E.coli*
**Otto et al. ** [15], **2011**	Parallel RCT Location: Australia	29 N (14 T/15 C)	18–40	Healthy adults	HBC with sodium bicarbonate (400 mg three times a day)	Lactose	7 days	Diarrhea, abdominal pain, and isolation of *E.coli* in stool	None	HBC was significantly effective in protecting against the development of diarrhea caused by ETEC (volunteers with diarrhea, 14% vs.86%; *p* = 0.0004) and lowering abdominal pain (0% vs. 36%;*p* = 0.04), but HBC had no significant effect on the number of diarrheal stools and the viability of *E.coli*
**Otto et al. ** [15],** 2011**	Parallel RCT Location: Australia	29 N (14 T/15 C)	18–40	Healthy adults	HBC without sodium bicarbonate (400 mg three times a day)	Lactose	7 days	Diarrhea, abdominal pain, and isolation of *E.coli* in stool	None	HBC was significantly effective in protecting against the development of diarrhea caused by ETEC (volunteers with diarrhea, 20% vs. 86%; *p* = 0.007) and lowering abdominal pain (0% vs. 36%; *p* = 0.02), but HBC had no significant effect on the number of diarrheal stools and the viability of *E.coli*
**Balachandran et al. ** [20], **2016**	Parallel RCT Location: Northern India	86 N (43 T/43 C) (48 M/38 F)	≤ 96 h	VLBW infants	Enteral BC (1.2–2 g four times a day)	Placebo	21 days	NEC occurrence, mortality and NEC clinical signs: abdominal distension, vomiting, pre-feed significant gastric residuals, blood in stool and ileus	None	BC supplementation showed no significant differences in the occurrence of NEC, mortality and NEC clinical signs: abdominal distension, vomiting, pre-feed significant gastric residuals, blood in stool and ileus
**Saad et al. ** [18], **2016**	Single-arm CT Location: Egypt	160 N (81 M/79 F)	1–6	Children with recurrent URTI and/or diarrhea	BC	-	4 wks	Episodes of diarrhea and frequency of hospitalization	Skin rashes, itching and diarrhea	BC significantly decreased episodes of diarrhea after 2 months (− 2.4; *p* < 0.001) and 6 months (− 2.2; *p* < 0.001) and number of hospital admissions (*p* < 0.001)
**Gaensbauer et al. ** [40], **2017**	Parallel RCT Location: Guatemala	301 N (154 T/147 C) (169 M/147 F)	6–35 months	Infants with acute non bloody diarrhea	BC and hyperimmune hen’s egg (7 g/d)	Hypoallergenic amino acid-based infant formula	3 days	Diarrhea duration	None	Combination of BC and hyperimmune hen’s egg had no significant effect on duration of diarrhea
**Eslamian et al. ** [17], **2018**	Parallel RCT Location: Iran	62 N (32 T/30 C) (35 M/27 F)	> 18	ICU-hospitalized patients	Enteral formula plus BC powder (20 g three times a day)	Isocaloric enteral formula plus maltodextrin	10 days	Abdominal distention, vomiting, diarrhea, constipation, and mortality	None	The incidence of diarrhea was significantly lower in BC group (9%) than in control group (33%), but there were no significant differences in abdominal distention, vomiting, constipation, and mortality at ICU between BC and control group
**Barakat et al. ** [48], **2019**	Parallel RCT Location: South Africa	160 N (80 T/80 C) (83 M/77 F)	6 months to 2 years	Children with acute diarrhea	BC plus standard therapy of acute diarrhea (3 g/d)	Placebo plus standard therapy of acute diarrhea	1 wk	Frequency of vomiting and diarrhea	None	BC group had a significantly lower frequency of vomiting (10% vs. 71.25%; *p* < 0.0001), diarrhea (0% vs. 12.50%; *p* = 0.001), and earlier time of disappearance of vomiting and diarrhea
**Rathe et al. ** [19],** 2019**	Parallel RCT Location: Denmark	62 N (30 T/32 C) (32 M/30 F)	1–18	Children with newly diagnosed ALL	BC (0.5–1 g/kg/d)	Isocaloric whole-milk powder enriched with whey protein isolate powder	4 wks	Intestinal mucositis, abdominal pain, and diarrhea	None	BC had no significant effect on intestinal mucositis, abdominal pain, and diarrhea
**Sanctuary et al. ** [35],** 2019**	Cross-over RCT Location: US	8 N (8 T/8 C) (7 M/1 F)	3.9–10.9	Children with ASD and GI comorbidities	BCP (0.15 g/lbw/d) + probiotics (*Bifidobacterium infantis*)	BCP only	5 wks	Constipation, diarrhea, pain, gas frequency, stool consistency, and gut microbiota	Gassiness and stomachache	BCP significantly reduced frequency of pain associated with bowel movements (-0.94; P = 0.044), diarrhea (-0.88; P = 0.021) and stool consistency (1.06; P = 0.042), BCP had no significant effect on gut microbiota
**Bierut et al. ** [41], **2020**	Parallel RCT Location: Southern Malawi	275 N (138 T/137 C) (162 M/113 F)	9 months	Healthy infants	BC (5.7 g) and dried whole egg powder (4.3 g twice daily)	Isoenergetic unfortified corn/soy flour (15 g)	3 months	Episodes of diarrhea and fecal microbiota	None	Combination of BC and egg supplementation had no significant effect on episodes of diarrhea and β-diversity of fecal microbiota

*M* male, *F* female, *n.d* not detected, *HBC* hyperimmune bovine colostrum, *BC* bovine colostrum, *BCP* bovine colostrum product, *HBCI* hyperimmune bovine colostrum immunoglobulin, *AIDS* acquired immunodeficiency syndrome, *BACI* bovine hyper immune anti-cryptosporidium colostrum immunoglobulin, *H. pylori Helicobacter pylori*, *UBT* C-urea breath test, *E. coli Escherichia coli*, *NSAID* non-steroidal anti-inflammatory drug, *HIV* human immunodeficiency virus, *SBS* short bowel syndrome, *VLBW* very low birth weight, *NEC* necrotizing enterocolitis, *URTI* upper respiratory tract infections, *ETEC* enterotoxigenic *Escherichia coli*, *ICU* the intensive care unit, *ALL*: acute lymphoblastic leukemia, *ASD* autism spectrum disorders, *GI* gastrointestinal

### Side effects

Nausea (8.1%) and flatulence (10.8%) [36], skin rashes (5.6%), itching (0.6%) [18], and increased gassiness (25%), and stomachache (12.5%) [35] were the reported side effects by studies participants.

### Risk of bias

**Fig. 2:**
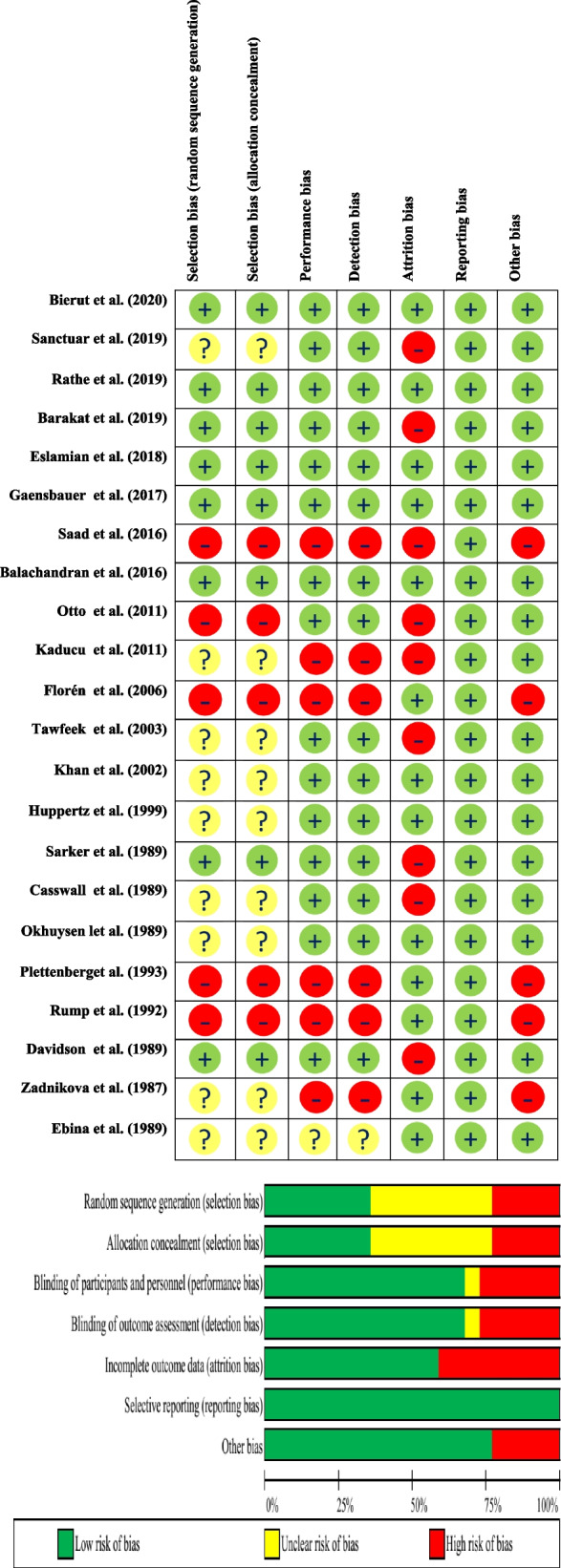
The methodological quality of included studies

The within-study risk of bias is summarized in Fig. 2. The Cochrane ROB tool for randomized control and cross-over trials was used to evaluate the risk of bias in the included studies [22]. For the selection bias domains (both randomization and allocation concealment), there were five studies with a high and nine studies with some concerns of bias, with the remaining studies (*n* = 8) with a low risk of bias. The inadequate generation of allocation sequences and allocation concealment are associated with biased intervention effects. In the domains of performance and detection biases, there was only one study with some concerns and six with a high risk of bias. The remaining 15 studies had low bias. Lack of blinding of participants or healthcare providers or outcome assessors could bias the results by affecting the actual outcomes of the individuals. Attrition bias was high in almost 50% of the included studies, while in the remaining ones, it was low. Differences between people lost to follow-up and those who continue can be the reason for any found effect and not the intervention itself.

All studies had a low risk of bias in the reporting domain. Five studies had a high risk of bias for the “other bias” domain, while the remainder had a low risk of bias (*n* = 17).

### Effect of intervention on outcomes

#### Diarrhea

Diarrhea was the most evaluated symptom in the included studies (*n* = 14) [15, 17–19, 26, 28, 30–32, 35–39]. Out of 20 effect sizes (extracted from 14 studies) examining the frequency or incidence of diarrhea, BC supplementation decreased diarrhea frequency in 15 intervention groups [14, 15, 17, 26, 28, 30–32, 35, 36, 38]. In contrast, no beneficial effect was reported in five interventional groups [19, 26, 30, 37]. BC could not shorten diarrhea duration in most studies [26, 35, 39] except for two [26, 28].

#### Stool frequency/evacuations

Stool frequency/evacuations were also examined in seven trials (8 effect sizes), all consistently suggesting a reduction [24–28, 32, 34]. BC supplementation, either with probiotics or alone, also improved stool consistency [35]. BC did not affect diarrheal stool output in four intervention groups [15] conducted among healthy adults but reduced it in children with rotavirus diarrhea [45], without any significant improvement in virus shedding in the stool [39].

#### Abdominal pain

Bowel movements or abdominal pain associated with bowel movements and abdominal distention were also examined by six studies (9 interventional arms) [15–17, 19, 20, 35, 36]. BC could alleviate abdominal pain in five interventional groups [15, 16, 36] but not in the remaining four articles [17, 19, 20].

#### Others

Other symptoms were poorly studied. These limited findings demonstrated that BC supplementation could decrease the presence of pathogens in stools [33, 34], improve bowel symptoms score (defined as patients’ well-being, abdominal pain, rectal bleeding, anorexia/nausea, bowel frequency, stool consistency, abdominal tenderness, and the presence of extra-intestinal manifestations), and inflammation [16] and vomiting frequency [48]. No beneficial effect of BC on *H. pylori* infection [34], the occurrence and clinical signs of NEC including abdominal distension, vomiting, pre-feed significant gastric residuals, blood in stool and ileus [20], severity of intestinal mucositis [19], gut microbiota [35], constipation [17], gastric residuals [20], blood in stool [20], ileus [20], and β-diversity of fecal microbiota [41] was also reported (Table 2).

**Table 2:** Effects of intervention on outcomes

	Inverse significant (favorable effect)	Non-significant
Bowel movements	[14–17, 35–39, 42, 44–48]	[17, 19, 35, 39, 40, 44, 47, 48]
Pathogen	[28, 43, 45, 47]	[15, 34, 35, 39, 41, 47, 48]
Inflammation	[16]	[19, 20]
Upper GI	[16, 48]	[20, 48]
Pain	[15, 16, 35]	[17, 19, 20]
General outcome (mortality and frequency of hospitalization)	[18]	[17, 43, 48]

Moreover, two studies examining the effect of BC on deficient birth weight and ICU-hospitalized patients failed to find any beneficial effect of BC on mortality [17, 20]. Despite a reduction in hospital admission frequency in children with diarrhea [18] following BC consumption, no reduction in the mean length of hospital stay was observed in hospitalized infants [43].

#### Hyperimmune BC

Five studies (including six trials) evaluated the effect of HBC [9, 18, 29, 30, 35]. These studies were conducted among infants and children but not one [44]. These studies revealed controversial results for the effect of BC on GI symptoms.

While HBC could decrease diarrhea incidence in infants [39] and tended to decrease it in adults [44], no improvement in the diarrhea duration was observed [39]. In the two other trials that examined the effect of monovalent and polyvalent HBC, only polyvalent HBC decreased diarrhea incidence, whereas monovalent HBC showed no significant reduction [47].

HBC also had a beneficial effect on rotavirus [43] but not on *H.pylori* infection [34].

There was debate about the effect of BC on GI symptoms based on BC dosage and duration of consumption.

## Discussion

Recently, the potential of BC as a therapeutic or nutraceutical product has been widely investigated. BC, also known as foremilk, has a nutrient profile and immunological composition, including nutritional factors, immunoglobulins, cytokines, growth factors, nucleosides, oligosaccharides, and antimicrobial agents [49]. Unlike human colostrum, which is enriched with IgA, BC predominantly contains IgG. To evaluate the efficacy of BC, it is essential to report the properties of its active components, such as the concentrations of IgG or antibody titers [50]. Supplementing BC may modulate immune responses, which improves symptoms of gastrointestinal tract disorders, especially inflammation, ulceration, and diarrhea. It seems that the anti-inflammatory effect of BC in GI epithelial cells is based on the suppression of nuclear factor-κB expression [51].

Although numerous BC products exist, details on their origin, extraction, manufacturing process, and standards must be better described, and analytical information is frequently missing from publications [52]. The information given in this review on the impacts of BC on GI diseases illustrates some of these problems.

In the current systematic review, 22 studies were identified which were heterogeneous in terms of their methodology, dosage, and preparation of BC, outcomes, and populations. Diarrhea is one of the GI diseases, which is among the leading causes of mortality and morbidity in the world and inflicts a tremendous health burden on children. Available evidence suggests the beneficial effects of BC supplementation on improving diarrhea in infants and patients [53].

Our systematic review showed that BC supplementation has the potential to improve diarrhea frequency, as well as stool evacuation/ consistency but not the duration of diarrhea [14, 15, 17, 26, 28, 30–32, 35, 36, 38]. Additionally, colostrum was found to be effective in reducing the frequency of hospital admissions due to diarrhea in children but not in reducing the length of hospital stay [18, 33]. Diarrhea can be caused by a combination of factors, including exposure to pathogens, the host’s immune system, and environment. Recent evidence indicates that BC supplementation may improve intestinal permeability and integrity, impacting diarrhea improvement [53]. The most common pathogens that cause diarrhea include rotavirus, coronavirus, enterotoxigenic *Escherichia coli* (*E. coli*), *Cryptosporidium parvum* (*C. parvum*), *Salmonella* spp., and *Clostridium perfringens* [54]. Our review showed that BC supplementation could improve bacterial (*E. coli*) and viral (rotavirus) infections related to diarrhea [33, 34]. Hence, BC may prevent or treat infectious diarrhea [55]. Since the gut is the epicenter of antibiotic resistance, this therapeutic approach is getting more attention from the research community to fight against bacterial infection of GIT than antibiotic-based medication.

Moreover, our systematic review has demonstrated that HBC has beneficial effects on diarrhea [9, 18, 34, 39, 44]. The immunization of cows produces HBC during pregnancy, which has a high level of antigen-specific IgG. HBC has been investigated to treat several enteric pathogens like *E. coli*, rotavirus, and *H. pylori* [51]. Two studies have investigated the quality parameters of HBC and found that only polyvalent HBC could reduce the incidence of diarrhea [47]. Polyvalent HBC may inhibit intestinal lipopolysaccharide (LPS) absorption. BC polyvalent immunoglobulins can also increase interleukin (IL)-10 and 13 and anti-inflammatory cytokine expression [56].

Our systematic review showed that HBC conferred a beneficial effect on acute rotavirus infection [33]. It is suggested that the vaccination of cows with uropathogenic *Escherichia coli* can stimulate a targeted immune response [57], which could be an interesting area for future studies. The specific type of HBC, with neutralizing titer activity against *H. pylori*, appears to have clinical utility in inhibiting the binding of *H. pylori* to lipid receptors [51]. In rodent models, HBC has been successfully effective in the reduction of *H. pylori* bacterial load [58]. However, our review did not find any beneficial effect of HBC on *H. pylori* infection [29], despite evidence showing that BC can inhibit the adhesion activity of *H. pylori* [29]. More studies are required to clarify whether HBC can be useful for eradicating *H. pylori*.

There is some evidence about BC’s anti-nociceptive activities [59]. The reduction in the frequency of bowel movement, abdominal distention, and pain associated with bowel movements following BC consumption was observed in three studies [15, 16, 35]. It has been demonstrated that colostrum contains several bioactive components that can influence the inflammatory process and antimicrobial activities and maintain intestinal immune balance [60]. The use of BC in treating IBD has been identified in just one study in which colostrum enemas ameliorated symptoms of left-side colitis [16]. Future trials should clarify the impact of oral consumption of colostrum in patients with IBD. Up to now, the therapeutic approaches for patients with IBD are still insufficient, and there is a need for alternative treatment options with novel mechanisms of action, like colostrum [61, 62]. Most benefits of BC in IBD seem to derive from its immune-modulating capabilities [24, 25].

Passive immunization is a logical alternative approach to protection from infectious diseases [63]. On a theoretical basis, it is expected that BC can balance and maintain intestinal microbiota. However, no beneficial effect of BC was reported on the β-diversity of fecal microbiota or intestinal microbiome [36, 37]. Additional research focused on the impact of BC on gut microbiota may be needed to confirm these findings.

Minor adverse reactions, such as nausea and flatulence [36], skin rashes/itching [14], and increased gassiness and stomachache [35], have been reported with the use of BC. Also, BC and HBC have been well tolerated. Therefore, BC is a valuable treatment for controlling gastrointestinal diseases with fewer side effects.

## Strengths and limitations

Up to now, there is a limited number of RCTs with small sample sizes to evaluate the effects of BC on GI health or diseases. The major limitation of this systematic review is the need for studies on this subject and differences in BC doses, outcomes, and study populations, which did not allow us to perform a meta-analysis in this regard. The lack of unpublished evidence may also be another limitation of this systematic review. Furthermore, most of the included studies had a high risk of bias, which seriously weakens confidence in the results. This paper is a comprehensive systematic review evaluating various GI symptoms following BC consumption. In addition, no restriction on the language and date of publication was made.

## Conclusion

This systematic review indicated that the nutraceutical approach of BC could improve the treatment of patients with diarrhea, inflammation, and bowel diseases.

## Future lines of research

Further RCTs should be conducted to support the benefits or potential contraindications of BC application in different GI diseases and outcomes to facilitate critical appraisal and interpretation.

Since the quality of BC is affected by many different factors, such as the health status of the cow, mammary glands, the season of the birth, gestation cycle of the cow, health management of the dairy product, and quality of diet during the dry period before parturition in cows, future research should be conducted to shed light on the effects of these factors on gut health. Future research projects are warranted to focus on the BC optimum dose and duration of supplementation. It is also suggested to find possible mechanisms underlying the effects of BC on GI diseases.

### Supplementary Information

**Additional file 1.** Search strategy.
