# Supplementary material for: Therapeutics effects of bovine colostrum applications on gastrointestinal diseases: a systematic review
Source: Syst Rev. 2024 Feb 26;13:76. doi: 10.1186/s13643-024-02489-1 (PMC10898101; doi:10.1186/s13643-024-02489-1)
Supplement: Supplementary file 1 — Additional file 1. Search strategy. [file 13643_2024_2489_MOESM1_ESM.docx]

| In PubMed, Scopus and ISI web of Science | n |
| --- | --- |
| 1. colostrum |  |
| 1. colostrums |  |
| 1. bovine |  |
| 1. cow |  |
| 1. cows |  |
| 1. cattle |  |
| 1. (1 OR 2 OR 3 OR 4 OR 5 OR 6) |  |
| 1. “Disease, Gastrointestinal” |  |
| 1. “Diseases, Gastrointestinal” |  |
| 1. “Gastrointestinal Disease” |  |
| 1. “Gastrointestinal Disorder” |  |
| 1. “Functional Gastrointestinal Disorders” |  |
| 1. (8 OR 9 OR 10 OR 11 OR 12) |  |
| (7 And 13) **In PubMed** | 3697 |
| (7 And 13) **In Scopus** | 1431 |
| (7 And 13) **In ISI web of Science** | 1753 |

**PubMed:**(colostrum[all]  OR colostrums[all] OR bovine[all]  OR cow[all]  OR cows[all]  OR cattle[all]) AND ((Disease[all]  AND Gastrointestinal[all]) OR (Diseases[all]  AND Gastrointestinal[all]) OR "Gastrointestinal Disease*"[all] OR "Gastrointestinal Disorders"[all] OR "Gastrointestinal Disorder"[all] OR "Functional Gastrointestinal Disorders"[all] OR "Functional Gastrointestinal Disorder"[all] OR ("Gastrointestinal Disorder"[all] AND Functional[all]) OR ("Gastrointestinal Disorders"[all] AND Functional[all]))

**Scopus:**

(TITLE-ABS(colostrum) OR TITLE-ABS(colostrums) OR TITLE-ABS(bovine) OR TITLE-ABS(cow) OR TITLE-ABS(cows) OR TITLE-ABS(cattle)) AND ((TITLE-ABS(Disease) AND TITLE-ABS(Gastrointestinal)) OR (TITLE-ABS(Diseases) AND TITLE-ABS(Gastrointestinal)) OR TITLE-ABS("Gastrointestinal Disease*") OR TITLE-ABS("Gastrointestinal Disorders") OR TITLE-ABS("Gastrointestinal Disorder") OR TITLE-ABS("Functional Gastrointestinal Disorders") OR TITLE-ABS("Functional Gastrointestinal Disorder") OR (TITLE-ABS("Gastrointestinal Disorders") AND TITLE-ABS(Functional)) OR TITLE-ABS("Cholera Infantum"))

**ISI Web of Science:**

(TS=(colostrum) OR TS=(colostrums) OR TS=(bovine) OR TS=(cow) OR TS=(cows) OR TS=(cattle)) AND ((TS=(Disease) AND TS=(Gastrointestinal)) OR (TS=(Diseases) AND TS=(Gastrointestinal)) OR TS=("Gastrointestinal Disease*") OR TS=("Gastrointestinal Disorders") OR TS=("Gastrointestinal Disorder") OR TS=("Functional Gastrointestinal Disorders") OR TS=("Functional Gastrointestinal Disorder") OR (TS=("Gastrointestinal Disorders") AND TS=(Functional)) OR TS=("Cholera Infantum"))
